# Supplementary material for: Transforming landscapes: Decoding the impact of universities on urbanization using advanced modeling and perception analysis
Source: PLoS One. 2024 Oct 16;19(10):e0302362. doi: 10.1371/journal.pone.0302362 (PMC11482722; doi:10.1371/journal.pone.0302362)
Supplement: S1 File — (DOCX) [file pone.0302362.s001.docx]

**Supporting Information (S1)**

Table A: Multicollinearity Test

| Variables | VIF | 1/VIF |
| --- | --- | --- |
| Age | 6.700 | 0.149 |
| Education | 6.240 | 0.160 |
| Land ownership | 1.760 | 0.568 |
| Income | 1.470 | 0.679 |
| Credit access | 1.370 | 0.728 |
| Distance from the university | 1.330 | 0.754 |
| Distance from Highway | 1.240 | 0.808 |
| Distance from Railway | 1.110 | 0.904 |
| Distance from hospital | 1.060 | 0.944 |
| Mean VIF | 2.470 |  |
