# Supplementary material for: Correction: Transforming landscapes: Decoding the impact of universities on urbanization using advanced modeling and perception analysis
Source: PLoS One. 2025 Mar 27;20(3):e0321234. doi: 10.1371/journal.pone.0321234 (PMC11949323; doi:10.1371/journal.pone.0321234)
Supplement: S1 File — (PDF) [file pone.0321234.s001.pdf]

PLOS ONE

RESEARCH ARTICLE

Transforming landscapes: Decoding the impact of universities on urbanization using advanced modeling and perception analysis

Md. Naimur Rahman[1,2,3]*, Md. Mushfiqus Saleheen[4], Babor Ahmad[5], Hamza El Fadili[6], Sagar A. S. M. Sharifuzzaman[7], Md. Salman Sohel[3], Shahnaj Husne Jahan[8], Md. Fouad Hossain Sarker[3], Abu Reza Md. Towfiqul Islam[3,9]*, Syed Anowerul Azim[4]

1 Department of Geography, Hong Kong Baptist University, Kowloon, Hong Kong, **2** David C Lam Institute for East-West Studies, Hong Kong Baptist University, Kowloon, Hong Kong, **3** Department of Development Studies, Daffodil International University, Dhaka, Bangladesh, **4** Department of Geography and Environmental Science, Begum Rokeya University, Rangpur, Bangladesh, **5** Department of Economics, Dhaka International University (DIU), Dhaka, Bangladesh, **6** Laboratory of Spectroscopy, Molecular Modeling, Materials, Nanomaterials, Water and Environment, Materials for Environment Team, ENSAM, Mohammed V University in Rabat, Rabat, Morocco, **7** Department of Intelligent Mechatronics Engineering, Sejong University, Seoul, South Korea, **8** Center for Archaeological Studies, University of Liberal Arts Bangladesh (ULAB), Dhaka, Bangladesh, **9** Department of Disaster Management, Begum Rokeya University, Rangpur, Bangladesh

* naimurbrur@gmail.com, naimurbrur@life.hkbu.edu.hk (MNR); towfiq_dm@brur.ac.bd (ARMTI)

Abstract

Universities play a crucial role in urban economic and structural development. The government of Bangladesh has undertaken the initiative to establish a public university in each of the 64 districts. These newly founded universities have the potential to impact urban growth significantly. We aimed to project university-induced urban expansion to address this knowledge gap and identify the mechanisms driving urban growth. The classification of supervised and unsupervised methods was employed to analyze urban development for the years 2000, 2010, 2016, and 2022. We used the Cellular Automata and Markov Chain approach to forecast future urban growth and land transition capacity. Additionally, the driving factors and selection of the study area were derived from Focus Group Discussions (FGD), Key Informant Interviews (KII), Probit Model, and Perception Index (PI). The findings of this study reveal a 1.6% urban growth rate within ten years of the establishment of the university, while urban expansion accelerated to 29.78% after ten years. The predictions also indicate a sustained urban growth rate of 4.7% by 2042. Furthermore, the PI index demonstrates that the establishment of the university has resulted in high demand for rental housing, serving as one of the primary drivers of urban expansion. Moreover, the Probit model highlights strong economic capability, proximity to the town, railway station, hospital, and easy access to credit as vital facilitators behind the drivers of urban expansion. Policymakers, the scientific community, and urban planners can benefit from this study in pursuing sustainable city development through university establishment.

OPEN ACCESS

Citation: Rahman M.N, Saleheen M.M, Ahmad B, El Fadili H, Sharifuzzaman SASM, Sohel M.S, et al. (2024) Transforming landscapes: Decoding the impact of universities on urbanization using advanced modeling and perception analysis. PLoS ONE 19(10): e0302362. https://doi.org/10.1371/journal.pone.0302362

Editor: Sotirios Koukoulas, University of Thessaly, School of Engineering, GREECE

Received: June 14, 2023

Accepted: April 3, 2024

Published: October 16, 2024

Copyright: © 2024 Rahman et al. This is an open access article distributed under the terms of the Creative Commons Attribution License, which permits unrestricted use, distribution, and reproduction in any medium, provided the original author and source are credited.

Data Availability Statement: All relevant data are within the paper and its Supporting Information files.

Funding: The work of C.A.D. was supported by the Delft Technology Fellowship grant awarded to G.G. by the Delft University of Technology. This work was also partially supported by the European Union (EU) through the ERC INSPIRE grant (101076926) to G.G.; views and opinions expressed are however those of the authors only and do not necessarily

reflect those of the EU or the European Research Council Executive Agency, and neither the EU nor the granting authority can be held responsible for them.

Competing interests: The authors have declared that no competing interests exist.

1. Introduction

Universities play a pivotal role in advancing educational, socio-economic, structural, cultural, and innovative outcomes [1, 2]. They significantly influence the urban living system, leveraging their extensive capabilities in knowledge and student facilities to drive technological and urban growth [3, 4]. Moreover, universities serve as catalysts for industrial, technological, and urban policy development, attracting investors and reshaping the growth patterns of specific regions [5]. These linkages contribute to cities' physical and environmental evolution [6]. The dynamics of urban expansion are time-dependent, and some universities are established in remote areas to improve socio-economic and structural conditions [7, 8]. Furthermore, universities located in urban or rural regions significantly influence spatiotemporal urban built-up dynamics.

While numerous studies worldwide have explored the social, economic, and cultural possibilities associated with universities [8, 9], the spatial-temporal urban growth resulting from universities remains less documented. For instance, Benneworth et al. examined how a university could enhance social and economic conditions by fostering mutual understanding among local authorities [10]. Kurucu & Chiristina argued that universities with high land density and property holdings significantly impact landscape development and micro-area urban growth [11]. Smith revealed that residential buildings, transportation networks, and local commercial opportunities directly result from university establishments and their effects [12]. These findings, such as those by Benneworth et al., provide crucial illustrations of how universities can lead to the structural development of suburban cities [10]. AYDIN analyzed the growth pattern of university students in relation to structural improvement and urban expansion dynamics [13]. In Bangladesh, universities also play a crucial role in developing a healthy society, economic progress, and cultural diversity [14]. Furthermore, the Government of Bangladesh (GoB) has decided to establish additional universities in the country's 64 districts [15]. As of 2022, the government of Bangladesh established 53 universities to meet the rising demand for higher education and promote regional development, aiming to enhance the nation's economic growth, employment, and social progress through a more accessible and diversified educational system. The establishment of universities in various districts also significantly impacts urbanization [16]. Therefore, investigating university-induced urban expansion and its future variability is essential for planning proper urban growth and developing sustainable cities.

Universities frequently contribute to the improvement of various sectors, including industrial, commercial, retail, social, economic, housing, and settlement growth [8, 10, 12, 13, 17]. They also promote sustainable urban life, ecology, ecosystem preservation, pollution control measures, and social cohesion. However, while university campuses often boast green spaces, they do not guarantee beneficial urban enhancements in their surrounding cities or remote areas. Therefore, it is crucial to determine the effect of universities on urban development outcomes. Various strategies have been employed in different studies to address this issue. For example, Rahman et al. used land use land cover (LULC), land surface temperature (LST), normalized difference vegetation index (NDVI), and urban heat island (UHI) approaches to investigate the effect of urbanization on surface temperature in seven districts of Bangladesh [18]. Other studies have also utilized LULC approaches to analyze urban dynamics [19, 20]. Han et al. employed unsupervised LULC classification to detect landscape changes [21]. Consequently, a range of modeling methodologies exists for studying urban expansion. Previous studies have predominantly used Logistic and linear regression [22–24]. However, in this study, we utilize Sentinel and Landsat imagery for expansion analysis and cellular automata (CA) and Markov chain (MC) modeling approaches. These methodologies have demonstrated

precision and accuracy in visualizing and modeling urban growth [25, 26]. Li et al. also employed the probit model, while You & Yang utilized the random forest model to extract driving factors [27, 28]. No study has investigated urban expansion trends using sentinel/landsat imageries and demonstrated future variabilities using CAMC models within a single study. Furthermore, this study is the first to employ the Probit model and Perception Index for identifying driving forces in examining urban expansion scenarios. These models have shown improved precision in various explorations [29–32].

Rangpur district is one of the urbanized regions of Bangladesh, experiencing rapid industrialization, commercial development, and agricultural expansion [18, 33]. Several studies have been conducted in the Rangpur district, such as Rahman et al. examining the effect of urban expansion on UHI [18], Roy et al. comparing LULC changes in two distinct cities [34], and Islam & Sarker focusing specifically on LULC changes in Rangpur City Corporation (RPCC) [35]. However, very few studies have investigated university-induced spatiotemporal urban growth and the extraction of its driving elements.

This study aims to comprehensively examine the influence of university establishments on urban expansion. Our primary objectives are to determine the extent of university-induced urban areas, identify the driving factors behind urban expansion, and predict the dynamics of urban growth. The investigation is designed to contribute to land management policy development, focusing on sustainable urban development and the enhancement of new university establishments. We introduce several novel contributions to the existing body of knowledge. Firstly, it examines the spatiotemporal urban growth resulting from universities, an aspect that has received limited attention in previous research. By investigating university-induced urban expansion and its driving forces, this study provides a comprehensive understanding of the impacts of universities on urban development. Furthermore, the utilization of Sentinel and Landsat imagery, combined with cellular automata (CA) and Markov chain (MC) modeling approaches, demonstrates a novel methodology for visualizing and predicting urban growth patterns. The intellectual merit of this study lies in its interdisciplinary approach, integrating aspects of urban planning, land management, and spatial analysis. By incorporating various data sources and modeling techniques, the study offers a robust framework for examining the complex dynamics of urban expansion and its underlying drivers. The broader impacts of this work extend to multiple stakeholders and sectors. Policymakers and urban planners can benefit from the insights gained through this study to inform decision-making processes related to sustainable city development, land management, and the establishment of new universities. Additionally, the findings contribute to the scientific community's understanding of the relationship between universities and urban growth, filling a gap in the existing literature. The study also has implications for the social and economic well-being of communities, as it addresses the potential impact of universities on local socio-economic conditions and cultural diversity. Overall, this study offers valuable insights into the role of universities in urban expansion, providing a foundation for informed decision-making and sustainable urban development strategies.

2. Study area

The study area is located within RPCC, encompassing Begum Rokeya University, Rangpur (BRUR) as shown in Fig 1. BRUR commenced its academic activities in 2009, and significant developments within our study area began in 2011. The choice of this area was pivotal for examining the impact of newly established universities on urban expansion, particularly in the context of recent urbanization trends. Our analysis focuses on the university's influence within a defined boundary of 7.30 square kilometers. This boundary was determined based on the

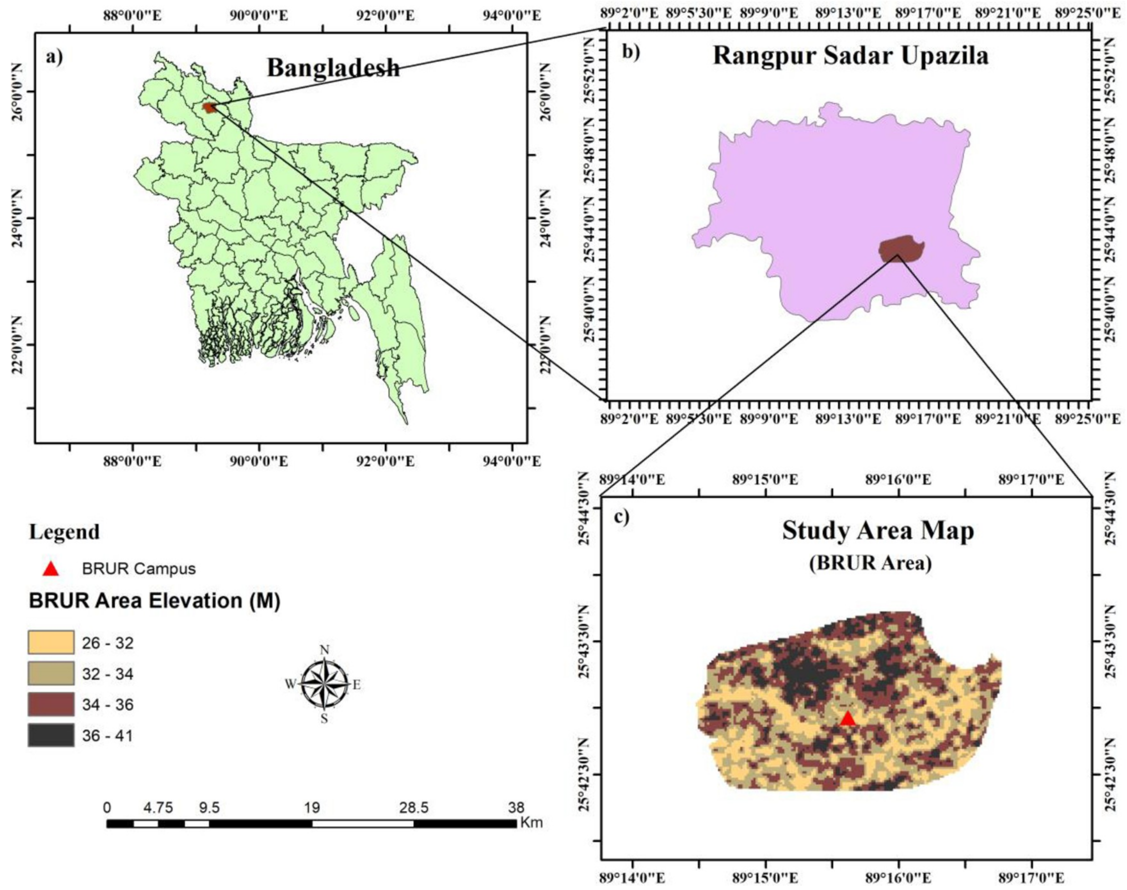

Fig 1. Study Area: a) Map of Bangladesh; b) Rangpur City Corporation; c) Final Study Area with Digital Elevation Model (source: author's own generated map, datasets of this map are obtained from a publicly and freely available site of United State Geological Survey (USGS), ref: www.usgs.gov).

https://doi.org/10.1371/journal.pone.0302362.g001

university's sphere of influence, employing Focus Group Discussions (FGD) and Key Informant Interviews (KII) for precise delineation, as depicted in Fig 2.

RPCC, including our study area, experiences a humid subtropical climate, characterized by an average temperature of approximately 24.92˚C and mean annual rainfall ranging from 2244 to 2498 mm, as reported by Rahman et al. and Rahman & Azim [18, 36, 37]. The population of Rangpur, as per the 2011 Bangladesh Bureau of Statistics (BBS) data, is approximately 796,556, with about 15% residing in urban areas [38]. The area's topography is relatively flat, with elevations varying from 26 meters to 41 meters above sea level, as depicted in Fig 1.

3. Data and methods

3.1 Focus Group Discussion (FGD)

This study was over-sighted via discussion with the people using convenient sampling Two gadgets were utilized to appraise the urban expansion areas due to the university establishment in the Rangpur district. Five FGDs were taken to the root level, including study areas in the north, south, east and west sides for compiling the assessment of the university-induced urban expansion boundary The author conducted FGD between 05 January to 20 February 2023. Each FGD has 12 members. A total of 60 participants were chosen for FGD, consisting of

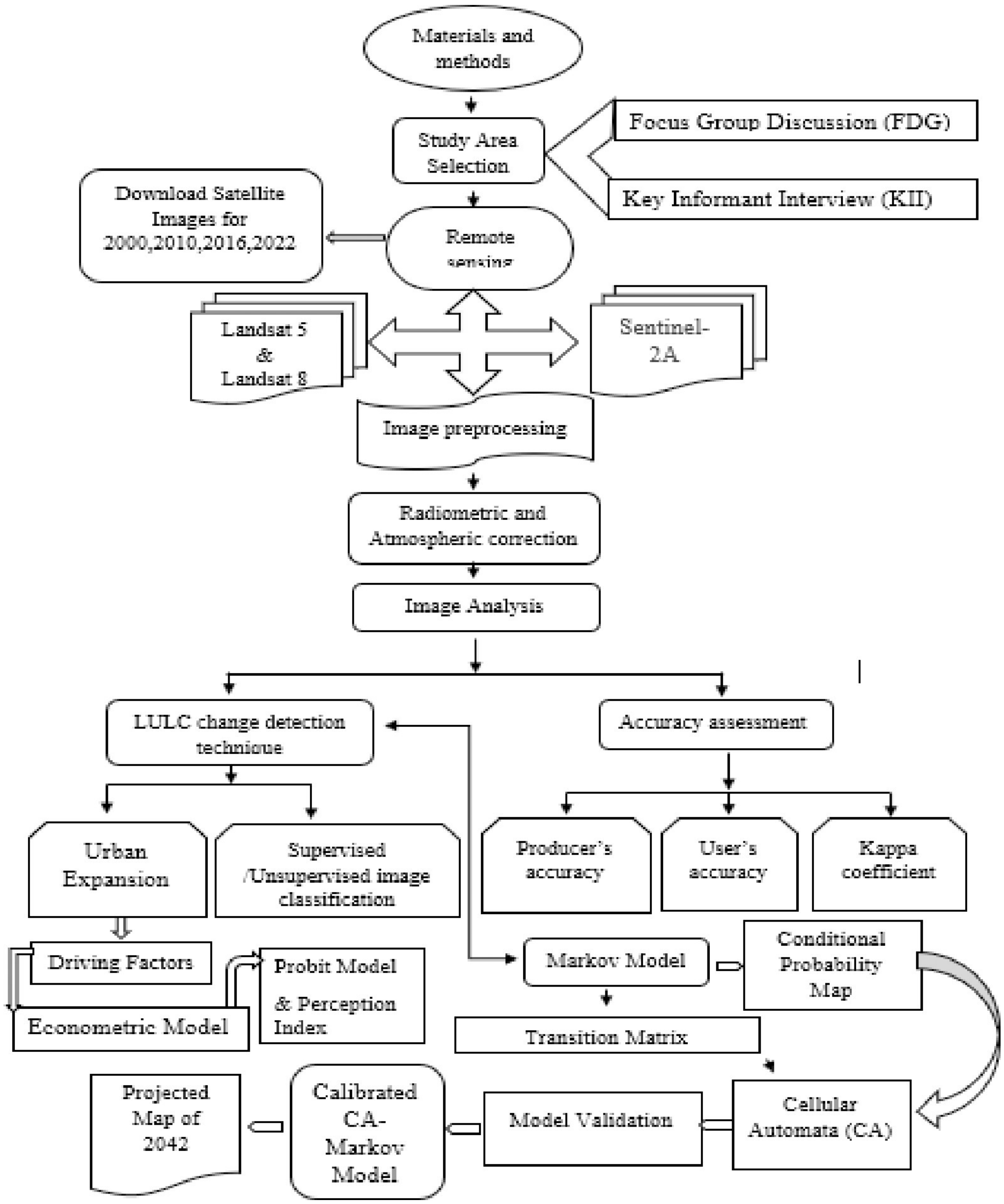

Fig 2. Methodological framework.

https://doi.org/10.1371/journal.pone.0302362.g002

diversified characteristics of the respondents (Bankers, House owners, Retired persons, Teachers of University and so on). The average age of the respondents was greater than 25 years old to abduct the actual scenario and actual university-induced urban areas by using their local experience and knowledge about the respective area. The following questions was usually asked to the participants in the discussions:

i. What is your general awareness of the establishment of universities in Northern Bangladesh and its potential impact on urbanization? How do you perceive the changes in urbanization and development in Northern Bangladesh over the past decade, particularly in areas where universities have been established?

ii. In your opinion, what role have universities played in the urbanization of Northern Bangladesh, and how do you think they have influenced the local communities?

iii. How has the establishment of universities affected the local community in terms of employment, education, infrastructure, services, and overall quality of life?

3.2 Key Informant Interview (KII)

Six groups of diversified personnel were selected randomly to capture their ideas and experience towards university-induced urban growth areas with KII, including i) locally social and politically influential persons (Male and Female), (ii) Planning level personals (Government Officers of LGED and LGD), iii)General public (Low income, middle income and high-income persons), iv) Agricultural Officers, v)Intellectuals (Teachers of university and colleges), vi) Research Workers (Research Associate of Begum Rokeya University, Rangpur).

The author purposively conducted 10 KII for achieving the study objective (Lindner & Dooley, 2008). Questionnaires regarding the locally influential and general personnel were done in the same alignment along with the socio-economic and geographical characteristics of the respondents. The following questions were frequently posed to discussion participants:

i. Can you provide an overview of the impact of universities on urbanization in Northern Bangladesh based on your knowledge and expertise?

ii. From your perspective, how have economic trends and employment opportunities evolved in the region with the growth of urbanization induced by universities?

iii. What role do universities play in the broader development of Northern Bangladesh, and how has this impacted the socio-economic landscape?

3.3 Data acquisition and pre-processing for satellite imageries

Two satellite imageries, Landsat and Sentinel, were obtained from United State Geological Survey (USGS) for 2000, 2010, 2016 and 2022 (Table 1). The Landsat images were collected at 30m resolution, where the sentinel was 10m. Therefore, cloud coverage remained within 10 percent for both images. For the analysis of LULC and its prediction, ArcGIS 10.8 and IDRISI Selva 17.02 was used. Hence for the driving factor analysis, STATA 17 version software was utilized. A detailed methodological framework can be sourced from (Fig 2).

3.4 LULC classifications

To explore the urban expansion scenario influenced by the university establishment, we employed LULC classifications. Therefore Landsat-7, Landsat-5 and Sentinel-2A imagery was used to identify four classes of LULC: water body, vegetation, grassland, and built-up areas. Also, for the LULC classes, we used a combined approach of supervised and unsupervised classification [39]. Additionally, the radiometric, atmospheric, and geometric correction was

Table 1. Obtained satellite image details.

Area	Year	Landsat/Satellite	Date of Acquisition	Sensor/ Instrument	Path and Row
University Influenced Study Area	2000	Landsat-7	17 November 2000	ETM	138/42
	2010	Landsat-5	05 November 2010	TM	
	2016	Sentinel-2A	17 October 2016	MSI	
	2022	Sentinel-2A	20 March 2022	MSI	

https://doi.org/10.1371/journal.pone.0302362.t001

performed before the final calculation of LULC classes [40]. Hence to accurately define Landsat data, we downscaled to a resolution of 20 meters, aligning them with the native resolution of Sentinel imagery.

3.5 Accuracy assessment

The accuracy assessment was conducted to test whether the classified maps were well suited to the ground data. We have analyzed 230 sampling points to validate our classified map [18, 41]. Thus, the validation was done through Google Earth Pro. The user, producer, and overall accuracy was investigated using the kappa coefficient through the error matrix [42, 43]. The accuracy result is considered in good agreement if the kappa coefficient is >0.75 [43, 44]. Hence this study found >0.75 kappa value for all the LULC classes where the classes from Sentinel images were more accurate (**Table 3**).

3.6 CA-MC model for LULC projection

The CA–MC approach has been extensively used to assess and quantify urbanized areas' growth and landscape changes. Additionally, it has proven its prediction capacity with greater accuracy [45]. The detailed working process of this model can be accessed from Lu et al. [46]. Therefore, two different year imageries (2000, 2016) were taken for calibration and optimization, while the 2022-year image was utilized to validate the prediction result. Finally, after performing the ground-based validation of the 2022 image, we predicted 2042 LULC dynamics.

3.7 Econometric analysis

Generally, a probit model is applied in modelling limited variable analysis or dummy variable outcome [47, 48]. This is used explicitly while the objective of the study is to analyze a latent variable say, denoted by y^* [48]. The excellence of the model is that it overcomes the shortcoming of the linear probability model and also able to predict the non-linear values of the explanatory. On the contrary, the basic frailty of the model is cannot expound the actual magnitudes of the parameters directly [49]. Probit model is considered for estimating the drivers of urban expansion. This is generally a type of binary regression. Binary Probit regression is applied here to identify the factors influencing the respondents toward urban expansion and its severity explained by Muthén [50]. This study uses this probit model due to its extensive acceptance in analyzing spatial data [51].

This study also uses the perception index (PI) introduced by Malhotra [52]. The whole scheme of estimating urban expansion drivers is depicted in Fig 3. This perception index analysis for the respondents' attitudes towards urban expansion will provide a clear doctrine which psychological phenomenon really matters for the respondents for associating with this development.

For estimating the parameters of the probit model, assume that p is a binary variable referring to less urban expansion due to university (m = 0) and high urban expansion (m = 1). Here, p is associated with a latent variable γ^*, which implies the proclivity of urban expansion engagement. The delineation betwixt p and γ^* remains as follows:

$$\{m = 0,\ if\ \gamma* \leq 0\ m = 1,\ if\ \gamma* > 0 \quad (1)$$

In Eq (1), γ^* can be gained using Eq (2) below.

$$\gamma^* = x\beta^T + \mu \quad (2)$$

Where $X = \{x_1, x_2, \ldots\ldots, x_n\}$ implies the vectors of the factors determining the severity of

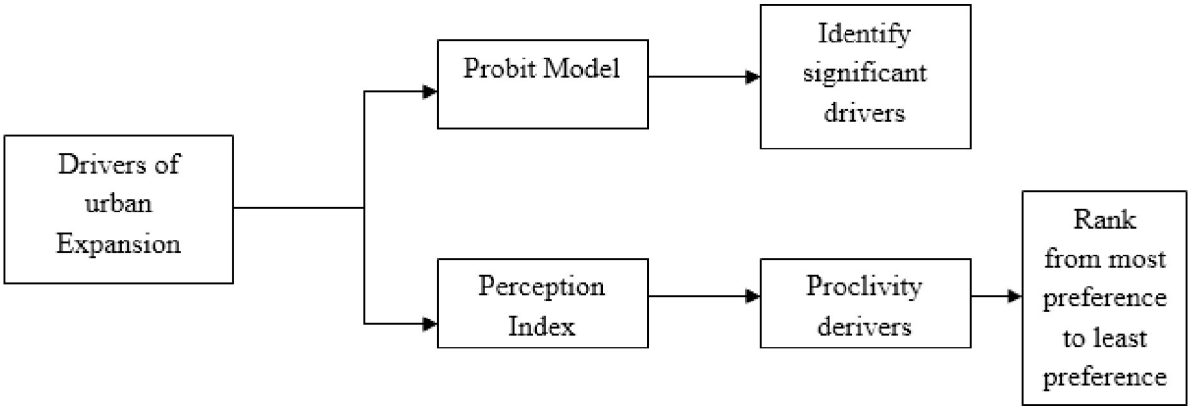

Fig 3. The scheme of the model of drivers for urban expansion.

https://doi.org/10.1371/journal.pone.0302362.g003

the urban expansion in the study areas. $\beta = \{\beta_1, \beta_2, \ldots\ldots, \beta_n\}$ is the respective unknown parameters to be estimated. μ refers to the stochastic disturbance term with a standard normal distribution ($\sigma = 1$).

Therefore, the possibility of having an urban expansion in the study areas can be obtained as below:

$$P(x) = P(x) = \varphi(x\beta^T) \quad (3)$$

Here, φ is the communicative distributional function which also follows $\sigma = 1$ (standard normal distribution). This study's marginal effect (ME) model is also used with mean. This procedure has been followed to find out that a unit change of any variable resulting from a unit change of another variable holds other things constant on the probability of $P(Y = 1| X = x)$. The selection of the driving variables is a challenging and difficult task as urban expansion and its growth varies from time to time as the intentions and attitudes of the inhabitants also [53]. Reviewing the past studies and opinion of virtuoso, nine explanatory variables have been selected, shown below in Table 2.

However, from the above nine variable: age of the respondents may be a key factor for the urbanization, this may be due to the fact that young people have better health status that may influence them to be more productive towards the urbanization related activities [54]. In addition, education as another socioeconomic ingredients have role on the urban socioeconomic

Table 2. List of the variables included in the probit model.

Variables	Meaning	Symbol	Nature of the variable
Dependent	Urban expansion = 1		
	No urban expansion = 0	Y	Categorical
Independent	Age of the respondent (Measured in years)	X_1	Continuous
	Educational status (Measured in years of formal Schooling)	X_2	Continuous
	Land Ownership (Measured in total capital assets)	X_3	Continuous
	Income (measured in BDT.)	X_4	Continuous
	Credit access (Dummy: 1 for yes and 0 for no)	X_5	Categorical
	Distance from the university (in kilometres)	X_6	Continuous
	Distance from the nearest Highway (in kilometres)	X_7	Continuous
	Distance from the nearest Railway (in kilometres)	X_8	Continuous
	Distance from the nearest hospital (in kilometres)	X_9	Continuous

https://doi.org/10.1371/journal.pone.0302362.t002

transformation also [55]. Education encourages persons to migrate from total to urban areas due to having better facilities which in turn affects the urbanization [55–57]. The cornerstones of urban development are coordinated urban operations including utilisation of land policy and land ownership. It has a bearing on the type and structure of growth in urban areas [58]. In addition to bestowing money and power upon its owners, land ownership also allows them to influence the policies and results of urban development. Finally, considering that ownership is a social construct and that metropolitan areas have an impact on ecological conditions, having land reflects the values of society [59]. Income and its distribution is another contribution factor for urbanization because an unequal distribution of the income causes slow growth of urbanization vice versa [60, 61]. Credit facility or availability of loan has increased the possibility of the agglomeration [62]. In case of health facilities, unplanned urbanization may create longer distance between the health communities and the inhabitants which in turn reflects the lower health facility and deprives the population from the benefit of urbanization [63]. Likewise, distance from the highway, railway and the university [64–67]. These mentioned reasons influence the researchers to include these explanatory variables in the probit model for accomplishing the objective.

4. Results

4.1 Accuracy

To evaluate the validation of four LULC classes, namely water body (WB), vegetation (VG), grassland (GL), and built-up (BA), accuracy was tested with ground data. Therefore, we found the highest accuracy for sentinel datasets of >94% (2016) and >97% (2022) images. However, good accuracy agreement is found for Landsat data, which includes 90% and 83.13% kappa values. Thus, user and producer accuracy are also significant for all the datasets (Table 3). However, sentinel data of 2016 and 2022 provides higher accuracy compared with Landsat imageries.

4.2 LULC dynamics and urban expansion

The four classes of LULC show significant changes, particularly for built-up areas in both spatial and temporal illustrations (**Fig 4 and Table 4**). The existence of the university is continuing since 2010. For this reason, from the beginning of 2000 to 2010 gradual increase in urban expansion is noticed, including >49 sq. km to >61 sq. km (**Figs 4, 5 and Table 4**). However, after the establishment of the university, rapid growth in the urban area is expanded from 2010 to 2016 of 8% to 17%, respectively. Therefore, it continues for 2022, includes of 38% extended urban areas. On the other hand, vegetation cover is significantly declined among different classes of LULC, indicating a 34% (2010) to 8% (2022) reduction. A fluctuating rise and fall is observed for waterbody from 2000 to 2016, but finally, it was found to be declined in 2022 (**Table 4**). Similarly, grassland also dropped at 53% (2022) from 56% in 2010 to 75% in 2000. The trend and spatial variation of the LULC indicates the dominance of grassland before

Table 3. Accuracy assessment.

Year	User Accuracy (%)				Producer Accuracy (%)				Kappa Statistics (%)
	WB	VG	GL	BA	WB	VG	GL	BA	
**2000**	94.60	93.34	85	81.82	100	87.5	80.95	100	90
**2010**	85.00	87.50	80.00	80.00	85.00	87.50	80.00	80.00	83.13
**2016**	97.50	85.00	90.00	92.50	100.00	89.47	100.00	88.72	94.25
**2022**	100.00	97.50	97.50	98.50	100.00	95.47	100.00	100.00	97.50

https://doi.org/10.1371/journal.pone.0302362.t003

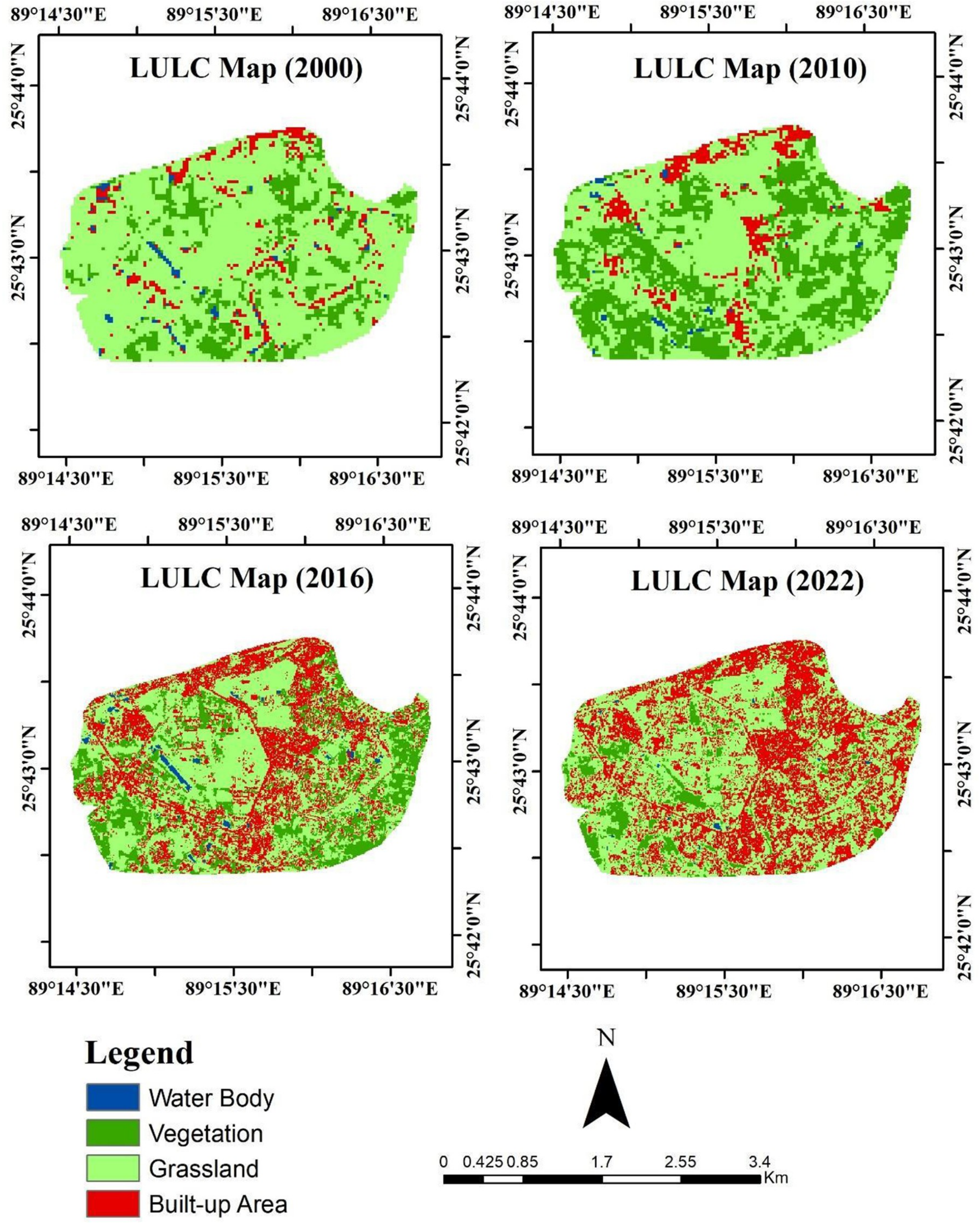

Fig 4. Spatial dynamics of LULC and urban expansion. (Source: author's own generated map, datasets of this map are obtained from a publicly and freely available site of United State Geological Survey (USGS), ref: www.usgs.gov).

https://doi.org/10.1371/journal.pone.0302362.g004

Table 4. Temporal dynamics of LULC and urban expansion.

LULC	Y2000		Y2010		Y2016		Y2022	
	sq.km	%	sq.km	%	sq.km	%	sq.km	%
WB	0.1125	1.541117	0.0621	0.850697	0.0877	1.201699	0.0191	0.261716
VG	1.1457	15.69474	2.529	34.64431	1.4996	20.54810	0.5931	8.126884
GL	5.5449	75.95857	4.095	56.09666	3.6719	50.31378	3.8991	53.42697
BA	0.4968	6.805573	0.6138	8.408334	2.0388	27.93642	2.7867	38.18443

https://doi.org/10.1371/journal.pone.0302362.t004

the establishment of BRUR 5.55 sq. km. in 2000 **(Figs 4 and 5).** Conversely, an extensive decline of grassland (>4 km^2) and vegetated land (>2.50 km^2) started after the university's establishment in 2010 and reached its highest peak in 2022 of >3.80 and >.50 km^2 **(Fig 2).** Hence the urban area is found in a significant upward trend from 2010(>.60 km^2) to 2022 (>2.75 km^2), which proves the influence of urban expansion caused by the university **(Figs 4 and 5).**

4.3 Temporal urban transition

The transition assessment provides the scenario of land conversion from one class to another. Significant urban expansion is noticed for all the transition periods of 2000–2022, 2010–2022, 2016–2022 **(Table 5).** Therefore, less conversion area is found from the water body to built-up area of 0.0126, 0.0081 and 0.0132 km^2 for the period of 2000–2022, 2010–2022 and 2016–2022 respectively. However, extensive conversion of LULC class into urban land is found for grassland and vegetated area. It includes 0.0441, 0.4257, and 0.4655 km^2 for vegetated land and 0.3924, 0.6084 and 1.0480 km^2 for grassland transition for three different periods. Moreover, heavy conversion of grassland and vegetated land is observed for the period of 2016–2021. In contrast, this noticeable urban area is converted into grassland for 2016–2022 of 0.75 km^2. Unlikely built-up area other LULC classes conversion is not significant except grassland conversion into the vegetation of 1.60 km^2 for 2000–2022.

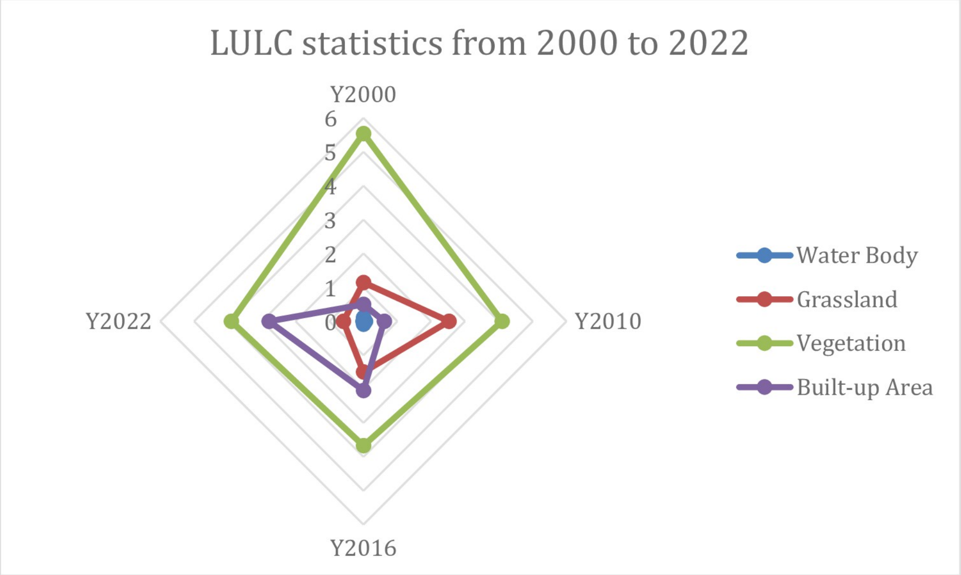

Fig 5. Trend of LULC dynamics.

https://doi.org/10.1371/journal.pone.0302362.g005

Table 5. Comparative assessment of urban temporal dynamics with different LULC classes.

Land Conversion	Change Transition	2000–2022	2010–2022	2016–2022
Land Converted Into Water Body	Grassland to Water Body	0.0162	00	0.004800
	Vegetation to Water Body	0.0018	00	0.000100
	Built-up Area to Water Body	0.0117	00	0.000600
Land Converted Into Grassland	Water Body to Grassland	0.0351	0.002700	0.057100
	Vegetation to Grassland	0.2439	0.1764	0.7889
	Built-up Area to Grassland	0.2736	0.0297	0.7481
Land Converted Into Vegetation	Water Body to Vegetation	0.0324	00	0.0038
	Grassland to Vegetation	1.5939	0.0252	0.3141
	Built-up Area to Vegetation	0.0468	00	0.0301
Land Converted Into Built-up Area	Water Body to Built-up Area	0.0126	0.0081	0.0132
	Grassland to Built-up Area	0.3924	0.6084	1.0480
	Vegetation to Built-up Area	0.0441	0.4257	0.4655

https://doi.org/10.1371/journal.pone.0302362.t005

4.4 Prediction

The transitional classes of landforms are categorized through Markov transitional matrix from 2000–2022 for three different periods (**Table 6**). Therefore, the value of the matrix periods varies from 0 to 1. In addition, it describes the probability that LULC classes will change to another category. A significant transition probability is found from all the classes to the built-up area for all the periods, especially from water body to built-up area of 0.73 and 0.71 probability for 2000–2022 and 2010–2022, respectively. After the built-up area highest possibility of transition is noticed for grassland for all the period. Therefore, a substantial positive probability is observed for 2016–2022 period from waterbody and vegetation to grassland, including 0.53 and 0.51 likelihood. However, two class of LULC is found in less agreement for transition: waterbody and vegetation. Thus, the lowest transition probability includes all the LULC classes to water bodies for all the periods (**Table 6**).

The urban expansion and other LULC classes were predicted for 2042 (**Table 7 and Fig 6**). Therefore, in facilitating this projection, a comprehensive land use change map has been compiled and is provided as Table A in **S1 File** for more detailed analysis and reference. Furthermore, the only positive upward trend is noticed for urban expansion includes 4.7% compared

Table 6. Transitional probability matrix.

	LULC	Period	To			
	Water Body	Vegetation	Grassland	Built-up Area		
From	Water Body	00–22	0.0000	0.0000	0.2652	0.7348
		10–22	0.0000	0.0066	0.2823	0.7111
		16–22	0.0021	0.0653	0.5264	0.4062
	Vegetation	00–22	0.0000	0.0229	0.2758	0.7013
		10–22	0.0000	0.0095	0.2818	0.7087
		16–22	0.0010	0.0617	0.5142	0.4231
	Grassland	00–22	0.0000	0.0215	0.2843	0.6941
		10–22	0.0000	0.0090	0.2819	0.7091
		16–22	0.0010	0.0622	0.5058	0.4211
	Built-up Area	00–22	0.0000	0.0685	0.2544	0.6770
		10–22	0.0000	0.0081	0.2818	0.7101
		16–22	0.0009	0.0566	0.5017	0.4408

https://doi.org/10.1371/journal.pone.0302362.t006

Table 7. Prediction of urban expansion includes annual dynamics of area and percentage.

LULC	2022(area)	%	2042(area)	%	Change in area (km)	Change %
Water Body	0.0191	0.26	0.0071	0.10	0.012	-0.16
Vegetation	0.5931	8.13	0.4371	5.99	-0.156	-2.14
Grassland	3.8991	53.43	3.7241	51.03	-0.175	-2.4
Built-up Areas	2.7867	38.18	3.1297	42.88	0.343	4.7

https://doi.org/10.1371/journal.pone.0302362.t007

with the 2022 change percentage. However, nearly the same decline of vegetation and grassland will be formed for the 2042 year of >-2%. Likewise, the decline of the water body will remain in 2042, of 0.16%. These findings clearly explain urban areas will be extended, followed by the declination of vegetation and grassland. The spatial visualization also follows the trend of urban expansion in all areas except some grassland in its west part (Fig 6). Hence, the outcome of the urban expansion probability and present changes is validated by **(Tables 4–7).**

4.5 Driving factors of university-induced urban expansion

The perception of the respondents regarding the drivers of urban expansion captured by using three scale Likert ranging from 1 to 3 which is presented in Table 8. This assessment is done

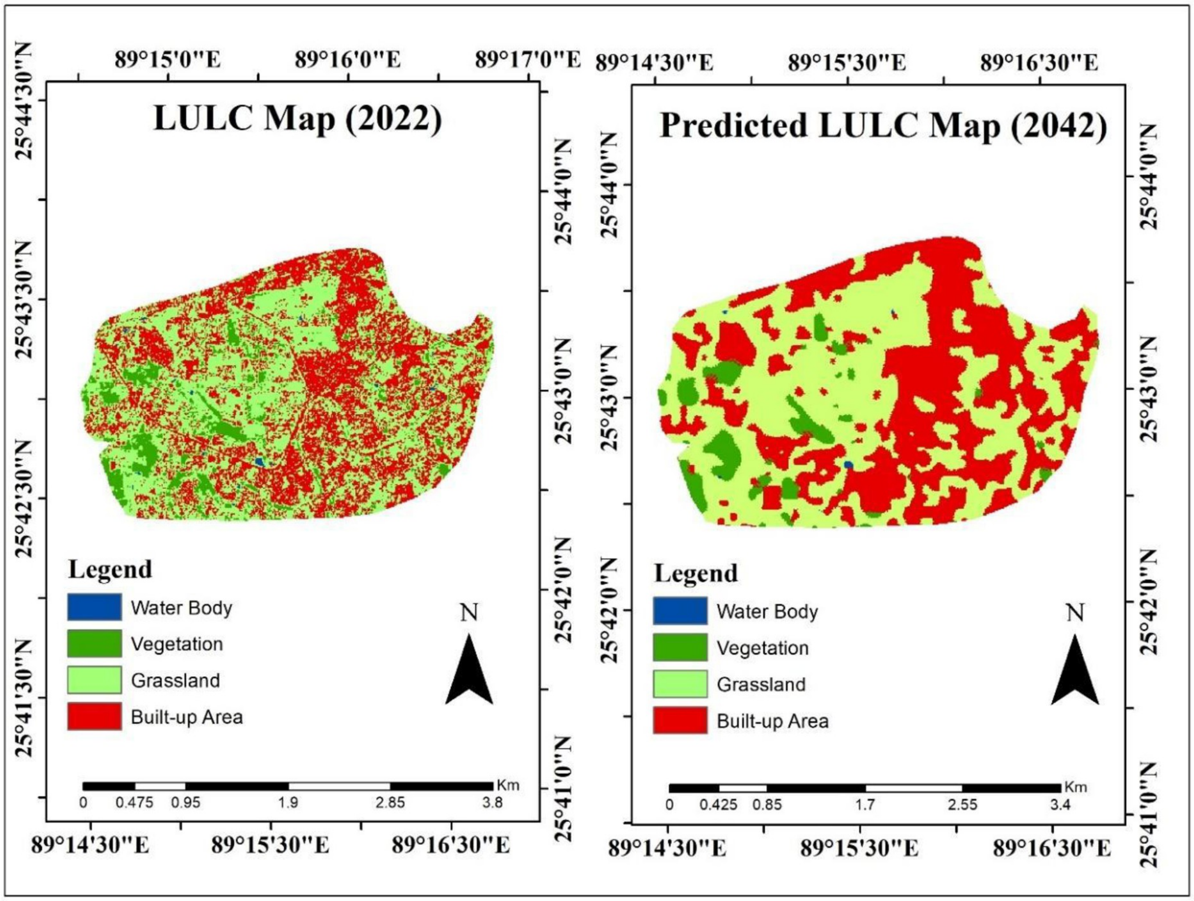

Fig 6. Present and projected spatial dynamics of LULC and urban expansion. (Source: author's own generated map, datasets of this map are obtained from a publicly and freely available site of United State Geological Survey (USGS), ref: www.usgs.gov).

https://doi.org/10.1371/journal.pone.0302362.g006

Table 8. Perception of the respondents regarding the urban expansion due to university.

Sl. No.	Statements	Nature of opinion			Perception Index	Rank
		Agree	Disagree	Neutral		
1.	Establishment of the university so that you can rent your house	53	0	5	169	1
2.	Open new business hub based on varsity	32	10	16	138	3
3.	Low price of land	19	24	15	111	7
4.	Coaching business based on varsity	11	13	34	127	6
5.	Location is near main town	45	5	8	156	2
6.	Easy accessibility of health facilities	09	36	13	71	8
7.	Transportation facilities	29	15	14	130	5
8	Education facilities	29	11	18	134	4

Source: Authors own calculation from field survey, 2022

https://doi.org/10.1371/journal.pone.0302362.t008

for figuring the most to less preferred reasons behind the adoption of urban expansion. Most of the respondents (likely 53) with PI of 169 agreed that they adopted urban expansion or their motive for movement towards urban expansion is the establishment of a university in that locality. This is because they can rent their house to staffs, students and teachers of that university which generates an additional source of income for them. This can't be ignored. The second important reason for the urban expansion in the study areas is the closest distance of town or local market for the urban expansion. This is another influential factor for urban expansion. The third influential determinant for driving people towards urban expansion is the openness of new business based on the university in the study areas, which is signified by PI of 138 and agreed by about 32. Educational facilities provided by the university ranked as 4th, expansion of transportation facilities or mode of communication ranked as 5th, the openness of coaching business based on varsity, including the openness of private coaching, tuition and other informal paid educational facilities ranked as 6th, lower price of land prevailing in the study areas for attracting investors ranked as 7th and easy accessibility of health facilities provided by both the local private medical clinic, hospitals and the governmental hospital ranked as 8th as the influential drivers of the urban expansion in that study areas.

4.5.1 Estimated result of probit model. Table 9 represents the outcome of the Probit model as the key determinant of the urban expansion in the study areas on average. Pseudo r-squared implies the goodness of fit for that model prevailing the study. Chi-square at 1% significant level reflects that the model is jointly highly capable of estimating the variables on average.

In the Probit model, age and educational qualification as socio-economic variables held by the respondent, land ownership pattern held by the respondent, monthly income of the respondent, and distance of the household from the university area are found as the significant drivers of the urban expansion of the study areas. Likewise, the marginal effect model also captures the result in the right panel of **Table 9**. The marginal effect model shows strong relation of income level, the establishment areas of the university closer to the household, distance from the railway, distance from highway road and distance from the nearest hospital with the urban expansion on average. In addition, the multicollinearity test of the nine variables was checked by the variance inflation factor (VIF) shown in Table A1. It has been seen that all the estimated result of the nine variables and the mean VIF is lower than enough to be stated as free from multicollinearity problem.

4.5.2 Marginal effect model. The second portion (Right side) of Table 9 portrays the result of the marginal effect of all the variables included in the study model. Basically, marginal

Table 9. Estimated result of probit model.

Variables	Probit model analysis			Marginal Effect Analysis		
	Coefficients	t-value	p-value	dy/dx	t-value	p-value
Age	0.176* (0.101)	1.74	0.082	0.018** (0.009)	1.980	0.048
Education	0.261 (0.546)	0.48	0.639	0.026 (0.054)	0.480	0.630
Land ownership	0.125* (0.059)	2.11	0.089	0.012*** (0.005)	2.730	0.006
Income	5.710** (1.748)	3.27	0.024	0.570*** (0.098)	5.790	0.000
Credit access	2.760* (1.496)	1.85	0.065	0.276** (0.136)	2.030	0.042
Distance from the university	-0.459** (0.223)	-2.06	0.04	-0.046** (0.019)	-2.360	0.018
Distance from Highway	-0.662* (0.376)	-1.76	0.079	-0.066** (0.034)	-1.950	0.051
Distance from Railway	-1.752* (0.933)	-1.88	0.061	-0.175** (0.083)	-2.100	0.036
Distance from hospital	-3.120** (1.543)	-2.02	0.043	-0.311** (0.135)	-2.300	0.021
Constant	-61.883*** (18.50)	-3.34	0.001			
Extra Statistics						
Pseudo r-squared	00.742					
Chi-square	60.489					
Prob > chi2	00.000					
Number of obs.	60.000					

ss in the parenthesis indicate standard errors; Asterisks refer *** p≤0.01

** p≤0.05

* p≤0.1

https://doi.org/10.1371/journal.pone.0302362.t009

effect model shows small response due to the change of explanatory variables with delta method. The bulkiest effect was crooked for a distance of the household from the university, railway, highway and hospital outlawed by the educational status of the household, land ownership and income of the household. Moreover, the larger distance of the household living place from the university area echoes decreases urban expansion by 0.046, which is statistically highly significant (p≤0.05). Similarly, an increase in age by one year decreased the urban expansion by 1.8% on average. This is also significant at 5% level. This may be because aged persons are more interested in urban expansion than the youth. In the case of income, a positive association has been identified in this study which implies that a 1% increase in the level of household income causes to increase in the urban expansion by 57% which is significant at 1% level and cannot be ignored. Therefore, a higher level of income has a crucial effect on urban growth. It can be concluded that areas with a larger number of high incomes have a higher rate of urban growth than those with a lower number of high-income groups.

Similarly, land ownership positively affects urban expansion, notifying that if the land ownership in decimals increases by 1%, it may lead to a 1.2% increase in the urban expansion in the study areas on average which is also statistically significant (p≤0.10). Additionally, credit access also has a significant (p≤0.05) positive influence on the urban growth on average

captured by 27.6%. This may be because people with higher credit facility have higher possibility to invest more on the urban expansion. This is identified as another crucial factor. Geographical factors such as distance from the nearest highway are negatively associated with urban expansion, which is statistically significant ($p \leq 0.05$). An increase in the study area distance from highway roads leads a reduction of urban growth by 0.066. Likewise, an increasing distance from the railway leads to 17.5% ($p \leq 0.05$) decrease in urban expansion, and a larger distance from the hospital causes 31.1% ($p \leq 0.05$) reductions in urban growth as a whole.

5. Discussion

Our study employed a comprehensive approach to evaluate the present urban expansion scenario in Rangpur, utilizing Landsat and Sentinel satellite images. We observed significant accuracy in the performance of Sentinel imagery over Landsat, consistent with findings by [68–70]. However, our use of Landsat 8 for the years 2000 and 2010 also demonstrated notable accuracy, as confirmed by Chowdhury et al. and Rahman et al. [18, 71]. In future studies, we aim to preferentially utilize Sentinel images. We employed Probit and Perception Index analyses to uncover significant results about the driving forces of urban expansion, which might encourage the combined future use of these models. We noticed an increase in urban expansion following the establishment of the university, likely driven by the housing needs of over 7000 students across 22 departments [72]. The university's presence has supported the development of various facilities and businesses, substantially validating our findings of urban expansion and the loss of vegetation and grassland. This phenomenon of university-influenced urban expansion echoes findings from similar studies in Turkey [73].

Our study also revealed that temporal transitions led to a reduction in vegetated areas while urban areas expanded, a trend supported by Kafy et al. and Roy et al. [44, 34]. Our prediction models suggest a dominant increase in built-up areas and a decline in grasslands by 2042, aligning with recent studies by Rahman et al. and Hasnahena et al. [18, 74]. Root causes of this urban sprawl, such as road transportation development and changes in land value, are consistent with findings by Harvey & Clark and Sudhira et al. [75, 76]. We assessed the drivers of urban expansion due to the university establishment and found that its location, along with proximity to the Central Business District and new business opportunities, were key influencers, as also noted by Xu et al. and Ollé & Marsal [77, 78].

Our econometric analysis using dummy variables and the Perception Index identified socio-economic and geographic factors as significant drivers of urban expansion, echoing findings from Huang et al. and Xu et al. [77, 79]. The study confirms that urban areas in Rangpur diminished with increasing distance from the university, a trend consistent with Quan & Tian and several studies emphasizing economic factors as primary influencers of urban expansion [77, 80–82]. However, geographical drivers such as proximity to the university or CBD had a larger impact than economic forces, aligning with the findings of Sarkar & Chouhan [53]. Our findings underscore the importance of location and education level in urban growth, highlighting the need for planned urban development and strategic policy formulation for future university establishments in Bangladesh.

The FGD and KII finding showed that establishment of universities correlated with a noticeable demand for housing and infrastructure. While participants acknowledged improvements, they also discussed challenges related to the rapid pace of development, particularly in terms of housing availability and affordability.

Our research on the impact of BRUR on urbanization reveals several unintended consequences. Key among these is the alteration of the housing market, where increased demand has escalated rental prices, benefiting property owners but potentially disadvantaging local

residents. Additionally, the local economy is undergoing significant shifts, with new businesses emerging to cater to the university community, which might sideline traditional local enterprises. Another crucial side-effect is the strain on existing infrastructure, including roads and public services, resulting from rapid urban expansion and population increase. This is accompanied by environmental impacts, such as reduced green spaces and increased pollution, and changes in social dynamics within the community.

These side-effects, while highlighted in the context of BRUR and Rangpur, are reflective of common trends observed in areas experiencing university-led urbanization. The aim of our study is not just to document these effects but to provide a nuanced understanding that can inform policy and planning. Acknowledging these side-effects is essential for developing comprehensive strategies that balance the positive aspects of university-induced development with measures to mitigate its less desirable consequences. Our findings are thus significant for their contribution to a more holistic approach to urban planning and policy formulation in the context of emerging urban centers in developing countries.

Our research provides a comprehensive analysis of the socio-economic and geographical distribution of university-induced urban development, as well as its driving forces and future variability. Therefore, the possible implications from this study could be the planned university establishment to contribute city development. Additionally, a policy may formulate based on the findings of future urban expansion and deriving factors. Consequently, a strategy for proper land utilization may be devised based on socio-economic perceptions and urban expansion outcomes. The limitation of the study includes only one university induced urban area is selected. Additionally, several universities from different district could bring different urban growth scenario and future changes.

5.1 Policy implications

The establishment of a university, particularly in a rural area, has the potential to provide several benefits to the local community. Some of the ways a university like this could improve the quality of life in rural areas. The presence of a university in a rural area can increase local residents' access to higher education, which can lead to better career opportunities and higher-paying jobs. This, in turn, can help to alleviate poverty and boost economic growth in the region. A university's presence can also attract businesses and investment to the area because it provides a pool of talented graduates who can be hired by local businesses. This can help to create new jobs and stimulate local economic growth. Furthermore, universities are frequently centres for research and development, which can aid in identifying and addressing local challenges and issues. A university in a rural area, for example, could conduct research on local agriculture or environmental issues and develop solutions to these issues. It is important to note, however, that the establishment of a university is not sufficient to ensure rural development. To facilitate easy access to the university and economic growth in the region, the government must also invest in infrastructure such as roads, transportation, and communication facilities. Furthermore, the government must ensure that the university is well-resourced and staffed with qualified educators in order to provide students with a high-quality education.

The rural university can also address urbanization challenges by focusing on issues such as urban-rural linkages, rural-urban migration, and the integration of rural and urban economies. For example, the university could conduct research on the effects of urbanization on rural communities and develop strategies to promote more balanced regional development. It can also provide education and training programs that help to develop urban-rural linkages, such as programs on sustainable urban agriculture, rural-urban entrepreneurship, and rural-urban planning.

Through outreach programs, community service, and partnerships, a rural university can engage with local communities. The university can help to build trust and strong relationships by collaborating closely with rural communities. This can help to foster a more collaborative approach to rural development and ensure that rural residents' needs are met. In summary, the rural-based university can play an important role in improving rural development by providing education, research, innovation, and community engagement that directly address the needs and challenges of rural communities.

6. Conclusion

In conclusion, this study has undertaken a comprehensive analysis of university-induced urban growth, employing a multi-faceted approach that includes the CA-MC model, FGD, KII, and questionnaire survey techniques. By concurrently utilizing the Probit and Perception Index methodologies, as well as introducing the CAMC model for urbanization projections, the research has successfully identified and predicted the factors driving urban expansion resulting from the establishment of a university.

The findings indicate a 1.6% urban growth rate within ten years of university establishment, accelerating to 29.78% after another ten years, with a sustained urban growth rate projected at 4.7% by 2042. The study also identified high demand for rental housing as a key driver of urban expansion, supported by the Probit model, which highlighted strong economic capability, proximity to essential facilities, and easy access to credit as vital factors. However, the probit model has a limitation that this does not consider the impact analysis of the water bodies (in distance), that is why this paves the ways for future study scope in broad concept.

Noteworthy implications for urban and regional planning and management arise from the study's predictions, particularly the decline in grassland and corresponding increase in built-up areas. The work contributes substantially to the understanding of how universities impact urban expansion, providing valuable insights for decision-makers involved in sustainable urban development, land management, and the planning of new university campuses.

The paper demonstrates a robust methodology by employing a multi-faceted approach that includes the CA-MC model, Focus Group Discussions (FGD), Key Informant Interviews (KII), and questionnaire survey techniques. This triangulation of methods enhances the accuracy and depth of the findings, providing a comprehensive understanding of the factors influencing urban growth following the establishment of universities.

However, an important consideration is the contextual specificity of the study's conclusions, primarily centered on the unique socio-economic and cultural landscape of Bangladesh. While this contextual depth strengthens the internal validity of the research within the Bangladeshi context, it introduces a potential limitation in terms of generalizability to other regions or countries. It is crucial for readers and policymakers to recognize this limitation, understanding that the study's insights may not universally apply to different settings.

Looking ahead, future research endeavours should consider conducting comparative studies across diverse regions and countries to assess the generalizability of these findings. Moreover, exploring the long-term effects of university-induced urban growth and its broader implications for social, economic, and environmental aspects will provide a more comprehensive understanding of this complex phenomenon. Incorporating advanced techniques such as remote sensing data and sophisticated modeling methods can further enhance the precision of urbanTop of Form.

Supporting information

S1 File. Multicollinearity test.
(DOCX)

S2 File. Dataset.
(XLSX)

Acknowledgments

We would like to acknowledge the respondent of our survey, the department of Geography and Environmental Science, Begum Rokeya University, Rangpur. Therefore we would like to further acknowledge Abdulla–Al Kafy for his valuable support in this research

Author Contributions

Conceptualization: Md. Naimur Rahman.

Data curation: Md. Mushfiqus Saleheen, Hamza El Fadili, Shahnaj Husne Jahan.

Formal analysis: Md. Naimur Rahman, Babor Ahmad, Hamza El Fadili, Sagar A. S. M. Sharifuzzaman, Syed Anowerul Azim.

Investigation: Md. Mushfiqus Saleheen.

Methodology: Md. Mushfiqus Saleheen, Hamza El Fadili.

Project administration: Md. Naimur Rahman.

Resources: Babor Ahmad, Syed Anowerul Azim.

Software: Sagar A. S. M. Sharifuzzaman.

Supervision: Abu Reza Md. Towfiqul Islam.

Validation: Babor Ahmad, Sagar A. S. M. Sharifuzzaman, Md. Salman Sohel.

Visualization: Md. Salman Sohel, Md. Fouad Hossain Sarker.

Writing – original draft: Md. Naimur Rahman.

Writing – review & editing: Md. Salman Sohel, Shahnaj Husne Jahan, Md. Fouad Hossain Sarker, Abu Reza Md. Towfiqul Islam, Syed Anowerul Azim.

References

1. Langford CH, Hall J, Josty P, Matos S, Jacobson A. Indicators and outcomes of Canadian university research: Proxies becoming goals?. Research policy. 2006 Dec 1; 35(10):1586–98.
2. Zhu C. Organisational culture and technology-enhanced innovation in higher education. Technology, Pedagogy and Education. 2015 Jan 1; 24(1):65–79.
3. Anselin L, Varga A, Acs Z. Local geographic spillovers between university research and high technology innovations. Journal of urban economics. 1997 Nov 1; 42(3):422–48.
4. YALÇINTAŞ A, Akkaya B. Türkiye'de akademik enflasyon:"her ile bir üniversite politikası" sonrasında Türk yükseköğretim sistemi. Ankara Üniversitesi SBF Dergisi. 2019 Jul 7; 74(3):789–810.
5. Ischinger B, Puukka J. Universities for cities and regions: Lessons from the OECD reviews. Change: The Magazine of Higher Learning. 2009 May 1; 41(3):8–13.
6. Yalcintan MC, Thornley A. Globalisation, higher education, and urban growth coalitions: Turkey's foundation universities and the case of Koc University in Istanbul. Environment and Planning C: Government and Policy. 2007 Dec; 25(6):822–43.
7. AKENGİN H KAYKI A. Şehirleşme üniversite ilişkisine bir örnek: Gazimağusa. Marmara Coğrafya Dergisi. 2013 Apr 10(28):501–25.
8. Compagnucci L, Spigarelli F. The Third Mission of the university: A systematic literature review on potentials and constraints. Technological Forecasting and Social Change. 2020 Dec 1; 161:120284.

9. Klofsten M, Fayolle A, Guerrero M, Mian S, Urbano D, Wright M. The entrepreneurial university as driver for economic growth and social change-Key strategic challenges. Technological Forecasting and Social Change. 2019 Apr Smith 1; 141:149–58.

10. Benneworth P, Charles D, Madanipour A. Building localized interactions between universities and cities through university spatial development. European planning studies. 2010 Oct 1; 18(10):1611–29.

11. Kurucu Y, Chiristina NK. Monitoring the impacts of urbanization and industrialization on the agricultural land and environment of the Torbali, Izmir region, Turkey. Environmental monitoring and assessment. 2008 Jan; 136:289–97. https://doi.org/10.1007/s10661-007-9684-4 PMID: 17370130

12. Atkinson R, Bridge G, editors. Gentrification in a global context. Routledge; 2004 Dec 10.

13. AYDIN İ. Demirci Şehri'nin mekânsal gelişimi ve gelişimi etkileyen faktörler. Marmara Coğrafya Dergisi. 2003(7).

14. Akther W. Contribution of universities in the implementation of SDGs: perspective Bangladesh. IOSR Journal of Business and Management. 2019; 21(9):21–7.

15. The Daily Star. (2018, January 14). Every district to get university. https://www.thedailystar.net/city/every-district-get-university-1520068

16. UGC. (2022). List of Public Universities. University Grants Commission of Bangladesh. http://www.ugc-universities.gov.bd/

17. Adamakou M, Niavis S, Kallioras D, Petrakos G. Understanding the regional engagement of universities from a stakeholders' perspective: The case of the University of Thessaly, Greece. Sustainability. 2021 Sep 23; 13(19):10565.

18. Rahman MN, Rony MR, Jannat FA, Chandra Pal S, Islam MS, Alam E, et al. Impact of urbanization on urban heat island intensity in major districts of Bangladesh using remote sensing and geo-spatial tools. Climate. 2022 Jan 4; 10(1):3.

19. Guo L, Liu R, Shoaib M, Men C, Wang Q, Miao Y, et al. Impacts of landscape change on net primary productivity by integrating remote sensing data and ecosystem model in a rapidly urbanizing region in China. Journal of Cleaner Production. 2021 Nov 20; 325:129314.

20. Zhang D, Xu J, Zhang Y, Wang J, He S, Zhou X. Study on sustainable urbanization literature based on Web of Science, scopus, and China national knowledge infrastructure: A scientometric analysis in CiteSpace. Journal of cleaner production. 2020 Aug 10; 264:121537.

21. Han J, Meng X, Zhou X, Yi B, Liu M, Xiang WN. A long-term analysis of urbanization process, landscape change, and carbon sources and sinks: A case study in China's Yangtze River Delta region. Journal of Cleaner Production. 2017 Jan 10; 141:1040–50.

22. Dewan AM, Yamaguchi Y. Land use and land cover change in Greater Dhaka, Bangladesh: Using remote sensing to promote sustainable urbanization. Applied geography. 2009 Jul 1; 29(3):390–401.

23. Dubovyk O, Sliuzas R, Flacke J. Spatio-temporal modelling of informal settlement development in Sancaktepe district, Istanbul, Turkey. ISPRS journal of photogrammetry and remote sensing. 2011 Mar 1; 66(2):235–46.

24. Vasenev VI, Stoorvogel JJ, Leemans R, Valentini R, Hajiaghayeva RA. Projection of urban expansion and related changes in soil carbon stocks in the Moscow Region. Journal of Cleaner Production. 2018 Jan 1; 170:902–14.

25. Deng J, Huang Y, Chen B, Tong C, Liu P, Wang H, et al. A methodology to monitor urban expansion and green space change using a time series of multi-sensor SPOT and Sentinel-2A images. Remote Sensing. 2019 May 23; 11(10):1230.

26. Xu L, Cui S, Wang X, Tang J, Nitivattananon V, Ding S, et al. Dynamic risk of coastal flood and driving factors: Integrating local sea level rise and spatially explicit urban growth. Journal of Cleaner Production. 2021 Oct 25; 321:129039.

27. Li G, Sun S, Fang C. The varying driving forces of urban expansion in China: Insights from a spatial-temporal analysis. Landscape and Urban Planning. 2018 Jun 1; 174:63–77.

28. You H, Yang X. Urban expansion in 30 megacities of China: Categorizing the driving force profiles to inform the urbanization policy. Land Use Policy. 2017 Nov 1; 68:531–51.

29. Azimi G, Rahimi A, Asgari H, Jin X. Severity analysis for large truck rollover crashes using a random parameter ordered logit model. Accident Analysis & Prevention. 2020 Feb 1; 135:105355. https://doi.org/10.1016/j.aap.2019.105355 PMID: 31812901

30. Bedon C, Fasan M. Reliability of field experiments, analytical methods and pedestrian's perception scales for the vibration serviceability assessment of an in-service glass walkway. Applied Sciences. 2019 May 11; 9(9):1936.

31. Hensher DA, Greene WH. The mixed logit model: the state of practice. Transportation. 2003 May; 30:133–76.

32. Zeng Q, Gu W, Zhang X, Wen H, Lee J, Hao W. Analyzing freeway crash severity using a Bayesian spatial generalized ordered logit model with conditional autoregressive priors. Accident Analysis & Prevention. 2019 Jun 1; 127:87–95. https://doi.org/10.1016/j.aap.2019.02.029 PMID: 30844540
33. Islam MR, Miah MG, Inoue Y. Analysis of land use and land cover changes in the coastal area of Bangladesh using landsat imagery. Land Degradation & Development. 2016 May; 27(4):899–909.
34. Roy B, Bari E, Nipa NJ, Ani SA. Comparison of temporal changes in urban settlements and land surface temperature in Rangpur and Gazipur Sadar, Bangladesh after the establishment of city corporation. Remote Sensing Applications: Society and Environment. 2021 Aug 1; 23:100587.
35. Islam W, Sarker SC. Monitoring the changing pattern of land use in the Rangpur City corporation using remote sensing and GIS. Journal of Geographic Information System. 2016; 8(04):537.
36. Rahman MN, Rony MR, Jannat FA. Spatiotemporal evaluation of drought trend during 1979–2019 in seven climatic zones of Bangladesh. Heliyon. 2021 Nov 1; 7(11). https://doi.org/10.1016/j.heliyon.2021.e08249 PMID: 34765774
37. Rahman MN, Azim SA. Spatiotemporal evaluation of rainfall trend during 1979–2019 in seven climatic zones of Bangladesh. Geology, Ecology, and Landscapes. 2023 Oct 2; 7(4):340–55.
38. BBS. (2011). Bangladesh Bureau of Statistics. Population and Housing Cencus.
39. Berihun ML, Tsunekawa A, Haregeweyn N, Meshesha DT, Adgo E, Tsubo M, et al. Hydrological responses to land use/land cover change and climate variability in contrasting agro-ecological environments of the Upper Blue Nile basin, Ethiopia. Science of the Total Environment. 2019 Nov 1; 689:347–65. https://doi.org/10.1016/j.scitotenv.2019.06.338 PMID: 31277003
40. Hishe H, Giday K, Van Orshoven J, Muys B, Taheri F, Azadi H, et al. Analysis of land use land cover dynamics and driving factors in Desa'a forest in Northern Ethiopia. Land use policy. 2021 Feb 1; 101:105039.
41. Talukdar S, Singha P, Mahato S, Pal S, Liou YA, Rahman A. Land-use land-cover classification by machine learning classifiers for satellite observations—A review. Remote Sensing. 2020 Apr 2; 12 (7):1135.
42. Cohen J. A coefficient of agreement for nominal scales. Educational and psychological measurement. 1960 Apr; 20(1):37–46.
43. Rwanga SS, Ndambuki JM. Accuracy assessment of land use/land cover classification using remote sensing and GIS. International Journal of Geosciences. 2017; 8(04):611.
44. Kafy AA, Faisal AA, Sikdar S, Hasan M, Rahman M, Khan MH, et al. Impact of LULC changes on LST in Rajshahi district of Bangladesh: a remote sensing approach. Journal of Geographical Studies. 2020 Jan 7; 3(1):11–23.
45. Fu X, Wang X, Yang YJ. Deriving suitability factors for CA-Markov land use simulation model based on local historical data. Journal of environmental management. 2018 Jan 15; 206:10–9. https://doi.org/10.1016/j.jenvman.2017.10.012 PMID: 29055845
46. Lu Y, Wu P, Ma X, Li X. Detection and prediction of land use/land cover change using spatiotemporal data fusion and the Cellular Automata–Markov model. Environmental monitoring and assessment. 2019 Feb; 191:1–9. https://doi.org/10.1007/s10661-019-7200-2 PMID: 30644019
47. Bishai D. Quality time: how parents' schooling affects child health through its interaction with childcare time in Bangladesh. Health economics. 1996 Sep; 5(5):383–407. https://doi.org/10.1002/(SICI)1099-1050(199609)5:5<383::AID-HEC225>3.0.CO;2-W PMID: 8922968
48. Kibria BG, Saleh AM. Improving the estimators of the parameters of a probit regression model: A ridge regression approach. Journal of Statistical Planning and Inference. 2012 Jun 1; 142(6):1421–35.
49. Damodar G. Basic Econometrics. Fourth Edition. Bright A, editor. The Economic Journal. New York: Gary Burke; 1972. https://doi.org/10.2307/2230043
50. Muthén B. A structural probit model with latent variables. Journal of the American Statistical Association. 1979 Dec 1; 74(368):807–11.
51. Cao Y, Zhang X, Fu Y, Lu Z, Shen X. Urban spatial growth modeling using logistic regression and cellular automata: A case study of Hangzhou. Ecological indicators. 2020 Jun 1; 113:106200.
52. Malhotra NK. Marketing research: an applied prientation. pearson; 2020.
53. Sarkar A, Chouhan P. Modeling spatial determinants of urban expansion of Siliguri a metropolitan city of India using logistic regression. Modeling Earth Systems and Environment. 2020 Dec; 6(4):2317–31.
54. Chen H, Liu Y, Li Z, Xue D. Urbanization, economic development and health: evidence from China's labor-force dynamic survey. International journal for equity in health. 2017 Dec; 16:1–8.
55. Raj J, Gopalkrishna MG. Role of education in urban socio-economic transformation. ZENITH International Journal of Multidisciplinary Research. 2020; 10(3):1–2.

56. van Maarseveen R. Urbanization and Education: The Effect of Childhood Urban Residency on Educational Attainment (Doctoral dissertation, Department of Economics, Uppsala University).
57. Knapp TA, White NE, Wolaver AM. The returns to migration: The influence of education and migration type. Growth and change. 2013 Dec; 44(4):589–607.
58. Khoei Z. The effects of land ownership system to urban development plan (case study: the section edge of Tehran City). International Journal of Architecture and Urban Development. 2018 Mar 1; 8(1):33–44.
59. Dixon T. Urban land and property ownership patterns in the UK: trends and forces for change. Land Use Policy. 2009 Dec 1; 26:S43–53.
60. Ros J. Development theory and the economics of growth. University of Michigan Press; 2001.
61. Ha NM, Le ND, Trung-Kien P. The impact of urbanization on income inequality: A study in Vietnam. Journal of Risk and Financial Management. 2019 Sep 10; 12(3):146.
62. Gabriel SA, Rosenthal SS. Urbanization, agglomeration economies, and access to mortgage credit. Regional Science and Urban Economics. 2013 Jan 1; 43(1):42–50.
63. Rahaman MA, Kalam A, Al-Mamun M. Unplanned urbanization and health risks of Dhaka City in Bangladesh: uncovering the associations between urban environment and public health. Frontiers in Public Health. 2023 Oct 19; 11:1269362. https://doi.org/10.3389/fpubh.2023.1269362 PMID: 37927876
64. Gregory IN, Henneberg JM. The railways, urbanization, and local demography in England and Wales, 1825–1911. Social Science History. 2010 Jul; 34(2):199–228.
65. Kasraian D, Maat K, van Wee B. Development of rail infrastructure and its impact on urbanization in the Randstad, the Netherlands. Journal of Transport and Land use. 2016 Jan 1; 9(1):151–70.
66. Mohammed AM, Ukai T. The impact of university campuses on city urbanization: A syntactic and socio-spatial analysis of Kyushu University campuses in Japan. International Journal of Sustainable Development and Planning. 2021; 16(7):1209–20.
67. den Heijer AC, Curvelo Magdaniel FT. Campus–city relations: Past, present, and future. Geographies of the University. 2018 Jul 31; 12(3):439.
68. Mishra PK, Rai A, Rai SC. Land use and land cover change detection using geospatial techniques in the Sikkim Himalaya, India. The Egyptian Journal of Remote Sensing and Space Science. 2020 Aug 1; 23(2):133–43.
69. Sánchez-Espinosa A, Schröder C. Land use and land cover mapping in wetlands one step closer to the ground: Sentinel-2 versus landsat 8. Journal of Environmental Management. 2019 Oct 1; 247:484–98. https://doi.org/10.1016/j.jenvman.2019.06.084 PMID: 31254763
70. Tavus B, Kocaman S, Gokceoglu C. Flood damage assessment with Sentinel-1 and Sentinel-2 data after Sardoba dam break with GLCM features and Random Forest method. Science of The Total Environment. 2022 Apr 10; 816:151585. PMID: 34767887
71. Chowdhury M, Hasan ME, Abdullah-Al-Mamun MM. Land use/land cover change assessment of Halda watershed using remote sensing and GIS. The Egyptian Journal of Remote Sensing and Space Science. 2020 Apr 1; 23(1):63–75.
72. BRUR. (2015). BRUR STUDENTS. https://brur.ac.bd/
73. Cetin M, Aksoy T, Cabuk SN, Kurkcuoglu MA, Cabuk A. Employing remote sensing technique to monitor the influence of newly established universities in creating an urban development process on the respective cities. Land use policy. 2021 Oct 1; 109:105705.
74. Sarker SC, Islam MS, Rahman MZ. The Rate and Pattern of the Spatio-Temporal Expansion of Rangpur City Corporation, Rangpur, Bangladesh. Current Journal of Applied Science and Technology. 2021 Jul 26; 40(17):74–83.
75. Harvey R. O., & Clark W. A. (1965). The nature and economics of urban sprawl. Land Economics, 41 (1), 1–9.
76. Sudhira H. S., Ramachandra T. V., & Jagadish K. S. (2004). Urban sprawl: metrics, dynamics and modelling using GIS. International Journal of Applied Earth Observation and Geoinformation, 5(1), 29–39.
77. Xu Q, Zheng X, Zhang C. Quantitative analysis of the determinants influencing urban expansion: A case study in Beijing, China. Sustainability. 2018 May 18; 10(5):1630.
78. Ollé AS, Marsal EV. Economic and political determinants of urban expansion: Exploring the local connection. Documents de treball IEB. 2007(5):1.
79. Huang QX, He CY, Shi PJ, Zhao YY, Yang Y, Liu HY. Understanding multi-scale urban expansion driving forces: In the case study of Beijing. Economic Geography. 2009 May; 29(5):714–21.
80. Quan Q, Tian G. A gradient analysis of spatiotemporal changes of urban landscape pattern in Beijing metropolitan region by GIS. Ecol. Sci. 2008; 27:254–61.

81. Li X, Zhou W, Ouyang Z. Forty years of urban expansion in Beijing: What is the relative importance of physical, socioeconomic, and neighborhood factors?. Applied Geography. 2013 Mar 1; 38:1–0.

82. Piao Y, Ma KM. Economic driving force of urban built-up area expansion in Beijing. Nat. Res. Econ. China. 2006 Jul; 7:34–7.
